# Skeletal Adaptations to Locomotion and Feeding in Mediterranean Batoids (*Raja asterias*, *Myliobatis aquila*) and the Teleost *Sparus aurata*: A Comparative Study

**DOI:** 10.3390/ani15203034

**Published:** 2025-10-19

**Authors:** Ugo E. Pazzaglia, Genciana Terova, Marzia Guerrini, Piero A. Zecca, Guido Zarattini, Fabrizio Serena, Cecilia Mancusi, Marcella Reguzzoni

**Affiliations:** 1 Department of Medical and Surgical Specialties, Radiological Sciences and Public Health, University of Brescia, 25121 Brescia, Italy; guido.zarattini@unibs.it; 2 Department of Medicine and Technological Innovation, University of Insubria, 21100 Varese, Italy; pieroantonio.zecca@uninsubria.it (P.A.Z.); marcella.reguzzoni@uninsubria.it (M.R.); 3 Department of Biotechnology and Life Sciences, University of Insubria, 21100 Varese, Italy; 4 Department of Chemistry, Physical Chemistry Section, C.S.G.I. (Consorzio Inter Universitario per lo Sviluppo dei Sistemi a Grande Interfase), University of Pavia, 27100 Pavia, Italy; marzia.guerrini01@universitadipavia.it; 5 Institute of Marine Biological Resources and Biotechnology, National Research Council (CNR-IRBIN), 91026 Mazara del Vallo, Italy; fabrizio50serena@gmail.com; 6 Environmental Protection Agency of Tuscany Region (ARPAT), 57126 Livorno, Italy; c.mancusi@arpat.toscana.it

**Keywords:** skeletal matrix mineralization, batoid locomotion, pectoral fin morphology, durophagy, teeth morphology, Chondrichthyes evolution

## Abstract

**Simple Summary:**

This study explored how the skeleton of the pectoral fins and feeding structures has evolved in two cartilaginous fishes (*Raja asterias* and *Myliobatis aquila*) and one bony fish (*Sparus aurata*). Our goal was to understand how these skeletal parts develop and adapt to different swimming styles and feeding needs. We found that the three species have distinct fin structures, which provide varying levels of flexibility suited to either open-water or bottom-dwelling movement. In *M. aquila*, we discovered two separate chewing systems: one external, using rows of teeth, and another internal, using special plates adapted for crushing hard prey (a feeding strategy known as durophagy). This dual system may allow the species to exploit a broader diet, or it could represent a remnant of an earlier evolutionary stage. Chemical analyses also showed that the minerals used to strengthen skeletal elements are the same in both cartilaginous and bony fishes, pointing to a shared evolutionary strategy. These findings provide new insights into how fish adapt their bodies to their environment and diet, offering a clearer picture of the evolutionary processes that shape survival in aquatic life.

**Abstract:**

In the Chondrichthyes *Raja asterias* and *Myliobatis aquila* and in the Teleost *Sparus aurata*, the appendicular skeleton of the pectoral fins (including the calcified structures of the mouth in *M. aquila*) was investigated to find out how the specific skeletal segments were formed and stiffened over the course of evolution, not only with regard to the adaptation of the ontogenesis of the cartilage “anlagen” to the mechanical requirements of locomotion in the water column, but also to the specific feeding habits (durophagy) of *M. aquila*. The morphology of the pectoral fins of the three species showed a different layout, characterized by the geometry of the basic units (aligned tesserae and calcified radial columns), which provide varied flexibility of the pectoral fins, suggesting an adaptation to the “pelagic” and “benthic” locomotion patterns in the environment where the species live. The morphology of the calcified structures in the mouth of *M. aquila* showed the presence of two different masticatory systems: the first (external) with the rows of teeth resting on the maxillary and mandibular arches, and the second (internal, in the oral cavity) with the symphyseal plates specialized for durophagy. Chemical–physical analyses revealed that the calcified cartilage matrix of the Chondrichthyes fin rays, teeth and durophagy plates is stiffened by the same Ca_3_(PO_4_)_2_ mineral phase deposed in the organic matrix of the Teleost *S. aurata* fins (with the characteristic SEM morphological texture of calcified bone matrix). The hitherto unknown presence of two different chewing systems in *M. aquila* documents an evolutionary adaptation to nutritional requirements that can be explained by two hypotheses: the coexistence of two functioning systems in current specimens, allowing for the ingestion of harder and softer prey (or plant food), or the persistence of a rudimentary dentition that is no longer used (vestigial dentition). Furthermore, the texture of the calcified matrix in teleost fishes, as observed by scanning electron microscopy, may indicate a bone-like organic matrix substrate, similar to that found in endochondral ossification.

## 1. Introduction

The skeleton of the Batoidea (Chondrichthyes), which includes both uncalcified and calcified cartilage, provides a unique insight into the evolutionary adaptation of vertebrate skeletal systems. In contrast to teleost fishes, tetrapods and marine mammals, calcified cartilage in Batoidea does not undergo endochondral ossification combined with the modeling and remodeling processes typically observed in fetal development and postnatal growth [1,2,3]. Instead, primary mineral deposition confers mechanical resilience to certain skeletal segments without subsequent replacement by a calcified bone matrix [3,4,5,6].

Among the batoid fishes, the species *Raja asterias* and *Myliobatis aquila* offer compelling opportunities for comparative analysis. Both species are native to the Mediterranean, but occupy different ecological niches and exhibit different movement and feeding behavior. These differences suggest that their skeletal architectures—particularly the attached elements of the pectoral fins and the structures associated with feeding—may have undergone different evolutionary adaptations to accommodate different mechanical requirements.

Recent discussions of morphological diversity within the Chondrichthyes have raised important questions about how developmental processes—particularly mineralization of cartilage—are modulated to support species-specific functions such as thrust generation, maneuverability, and hard-shelled prey processing [5,7,8,9]. In this context, the study of fin rays, jaws, teeth and specialized feeding adaptations such as durophagy provides crucial insights into the links between form, function and environment.

While many vertebrates transition from cartilaginous to bony structures through ossification, batoids rely exclusively on the primary mineralization of cartilage. This process provides the rigidity and resilience needed for their skeletal elements. These contrasting strategies raise an important question: how can different mineralization pathways produce functionally equivalent biomechanical outcomes? They also prompt us to ask whether convergent patterns of mineralization occur across different taxonomic groups, including teleosts and mammals.

Another compelling aspect concerns the feeding apparatus, where structural adaptations in the skull and oral cavity may reflect feeding specialization. In particular, the occurrence of internal oral plates in durophagous species such as *M. aquila* prompts the question of whether such features represent evolutionary novelties or modifications of ancestral traits. The possible coexistence of multiple masticatory systems in a single organism also invites reconsideration of established assumptions about the functional anatomy of chondrichthyans.

To address these questions, an integrative approach combining anatomical, histological and physicochemical analysis is essential. Such an approach enables a comprehensive understanding of how skeletal morphology and material composition relate to ecological role and evolutionary pressure. This study therefore aims to explore the adaptive morphological and mineralizing strategies in *R. asterias*, *M. aquila*, and *S. aurata* (the latter widening the comparison to the teleost skeleton) with particular attention to the structural organization of the pectoral fins and feeding apparatus. In doing so, it contributes to the broader discourse on the evolution of vertebrate skeletal systems and the diversity of solutions that have evolved in response to functional requirements.

## 2. Materials and Methods

### 2.1. Ethics Statement

The study protocol received approval from the Council of the Department of Medical and Surgical Specialties, Radiological Sciences, and Public Health at the University of Brescia (approval code: 171.3; approval date: 7 May 2015).

The material for this study was obtained from chondrichthyan fishes of the two species, *R. asterias* and *M. aquila*, collected during recent scientific research campaigns conducted by Tuscany ARPAT in the northern Tyrrhenian Sea, as well as from specimens that died naturally at the Livorno Aquarium (Livorno, Italy). In addition, two specimens of the teleost gilthead sea bream (*S. aurata*) were provided by the fish breeding facility of the University of Insubria. In total, seven specimens were examined (Table 1). All specimens were frozen within 12 h of capture or death in the aquarium and stored at −30 °C until photographs, measurements and dissections were performed. The fish were thawed by immersion in a 40 °C phosphate-buffered solution, after which the left and right pectoral and pelvic fins were separated from the head and girdles in chondrichthyan specimens, and the pectoral fins were removed from the body in *S. aurata*. Following photographic documentation, all specimens were fixed in 10% buffered formaldehyde for subsequent processing.

### 2.2. R. asterias and M. aquila Pectoral Fin Radiographs

*Raja asterias* and *M. aquila* pectoral fin radiographs were taken after manual dissection of the superficial muscle layers and ventral/dorsal skin, leaving a peripheral strip of about 20 mm of dorsal skin to avoid damage to the thin, most laterally located radialis.

(1) A first group of fin specimens was subjected to a more detailed muscle dissection. In particular, the specimen was immersed in a bath of 50% concentrated H_2_O_2_ solution for one week so that remnants of oxidized soft tissue on the surface could be removed with a toothbrush. The specimens were then rinsed in a phosphate-buffered solution and viewed in transmitted light under a microscope [3].

(2) Small samples of the fin skeleton were firmly enclosed between two glass slides, dehydrated in ascending concentrations of ethanol and heated in an oven at 300 °C for 12 h (heat deproteinization) and allowed to cool at room temperature. Some of these samples (after removal of the upper slide) were mounted on the lower glass slide with a cover slip to be viewed in transmitted light at low magnification under a light microscope [3].

(3) Other heat-deproteinated samples were detached from the slide, mounted on stubs with bi-adhesive carbon tape, and coated with a thin layer of gold using a sputter coater (Emitech, Montigny-le-Bretonneux, France). These preparations were examined with a ZEISS GeminiSEM 360 scanning electron microscope (Zeiss, Milan, Italy). The instrument operates with an accelerating voltage range of 0.02–30 kV and allows high-resolution imaging at a short analytical working distance of 8.5 mm, although the working distance can be adjusted depending on the application and desired resolution. In this study, surface fields were examined at magnifications of 150× and 350× on gold-coated samples to assess morphological features, while X-ray energy-dispersive spectroscopy (EDAX) was carried out on carbon-coated specimens to characterize the composition of the calcified cartilage matrix. The resulting spectra were then compared with those obtained from the calcified bone matrix of the pectoral fins of the teleost *S. aurata*.

(4) Additional fin samples were decalcified in a 2% acetic acid/2% hydrochloric acid solution for three weeks, dehydrated through a graded ethanol–water series, and embedded in paraffin. Sections 10 µm thick were cut, stained with hematoxylin and eosin, mounted on glass slides, and examined under an Olympus BX-51 microscope (Olympus Ltd., Tokyo, Japan).

Other fixed, undecalcified specimens were embedded in Technovit 7200 resin (Kulzer GmbH, Hanau, Germany). Sections 150 µm thick were prepared using the EXAKT cutting-grinding system (EXAKT Advanced Technologies GmbH, Norderstedt, Germany), stained with methylene blue–acid fuchsine, and observed with the same microscope.

### 2.3. The Head Photographs of R. asterias and M. aquila

The head photographs of *R. asterias* and *M. aquila* as well as the X-ray images in dorso-ventral and lateral projection of the two most similar specimens of the study group in terms of sex and weight were compared (Table 1). The head was separated from the rest of the fish body to allow radiographs to be taken in lateral and dorso-ventral projection. In addition, the head of *M. aquila* was dissected in more detail to separate the upper and lower symphyseal plates from the jaws and to obtain samples of appropriate size for micro-CT, histomorphology and mineral analyses.

Dry, undecalcified, 1 mm thick fin samples were thermally deproteinized in a muffle furnace at 400 °C for 24 h and mounted with coverslips for microscopic observation.

### 2.4. Chemical Mineral Analysis

Thermogravimetric analysis (TGA), Fourier transform infrared spectrometry (FTIR), X-ray diffraction (XRD) and EDAX (with SEM) were performed on the following samples: *M. aquila* symphyseal plates and radials; *R. asterias* maxillary cartilage; *S. aurata* pectoral fins.

Thermogravimetric curves were recorded using a thermogravimetric analyzer (TGA Q5000, TA Instruments Inc., New Castle, DE, USA) connected to the TA5000 data station by heating approximately 5 mg of each sample in a platinum crucible under N_2_ flow (50 mL min^−1^) from 25 to 1000 °C at 10 °C·min^−1^. The data were analyzed using Universal Analysis 2000 software from TA Instruments, also taking into account the derivative of weight versus temperature (DTG curve).

Infrared spectra were recorded at room temperature using a Nicolet FTIR iS10 spectrometer (Nicolet, Madison, WI, USA) equipped with a Smart ITR with diamond plate. Thirty-two scans in the 4000–600 cm^−1^ range at a resolution of 4 cm^−1^ were merged. X-ray powder diffraction analyses were performed by covering a moncrystal Si sample holder without background with a thin layer of the different samples and recording the pattern from 10° to 80° using a Bruker D8 diffractometer (40 kV and 40 mA for X-ray generation, Cu Kα radiation and Ni-filter, 0.02° step size, 10 s counting time).

## 3. Results

### 3.1. Macro- and Microscopic Morphology of the Pectoral Fins

**Figure 1:**
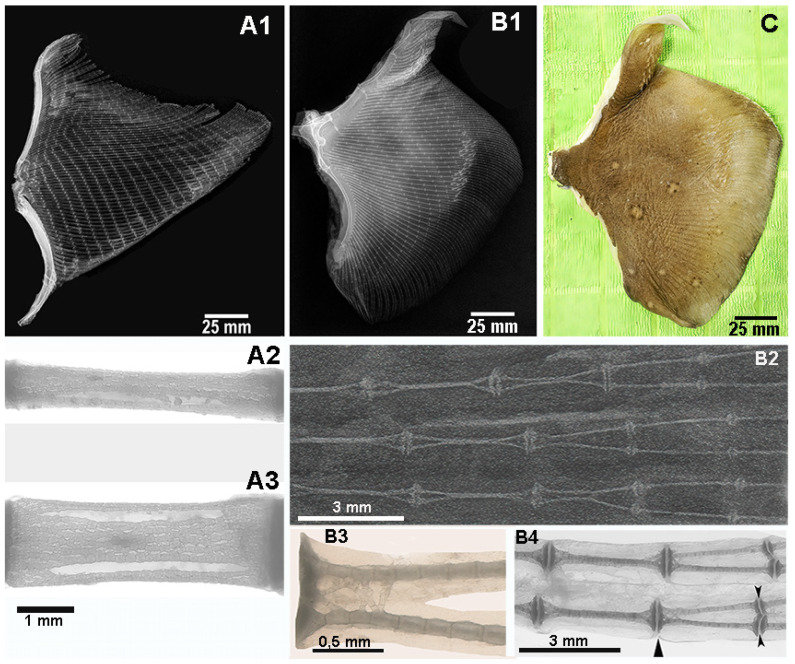
*Myliobatis aquila* and *Raja asterias* right pectoral fins. (**A1**) *M. aquila*: radiograph in the dorso-ventral projection of the right fin showing the rays consisting of aligned radials of similar length from the pterygia to the outer margin. (**A2**,**A3**) *M. aquila*: higher magnification radiographs of the radials in lateral and dorso-ventral projection showing the pattern of internal calcified cartilaginous columns. (**B1**) *R. asterias*: radiograph in dorso-ventral projection of the right fin showing the rays with aligned (vertically over-lapped), calcified columns in the medial fin sector. Rotation of the two vertical columns in the horizontal plane at about 2/3 of the ray’s length, in such a way doubling the number of single-column rays. (**B2**) *R. asterias*: higher magnification radiograph in dorso-ventral projection, documenting the rotation of the overlapping calcified columns in the horizontal plane. (**B3**,**B4**) *R. asterias*: trans-illumination images in dorso-ventral projection of the two inner, calcified cartilage columns. Details of the joint’s plates connecting the columns at the extremities. Splitting of the joint disk allows horizontal rotation of the vertically overlapped calcified columns (in the medial sector) and transformation of mono-columnar radial in the lateral fin sector. Black arrows in panel B4 indicate the joint disk splitting. (**C**) *R. asterias*: fin dorsal view showing the brown and rough dorsal skin, round speckles and denticles on the surface. Note: The different background tones between (**A2**), (**A3**) and (**B2**) result from the use of different X-ray machines for the two specimens.

The pectoral fins of *M. aquila* are triangular in shape, with the medio-lateral axis wider than the antero-posterior length of the fin base (i.e., the pterygial base, the skeletal foundation that supports the fin rays). In contrast, the fins of *R. asteria* are rounded and tapered anteriorly, with a continuous skin between the head and the rostrum (Figure 1(A1,B1)).

When comparing two specimens of *M. aquila* and *R. asterias* with similar body weights (Table 1), the most striking external difference was the dorsal pigmentation: *M. aquila* had a smooth, black dorsal surface, whereas *R. asterias* had a rough, brown dorsal surface characterized by two symmetrical, roundish spots (Figure 1C). Dermal denticles were clearly visible on the surface of the dorsal fin of *R. asterias*, as documented on dorso-ventral radiographs (Figure 1(B1)), whereas they were absent in *M. aquila*. The ventral skin was smooth and whitish in both species. Counting the rays in the left pectoral fin revealed 75 rays in *M. aquila* and 86 in *R. asterias* (Figure 1(A1,B1)).

In both species, each fin ray is composed of multiple elements called radials which are separated by inter-radial joints, i.e., amphiarthroses, that act as slightly movable joints. The joints between the girdle and the first radials line allow a movement in the dorso-ventral plane wider than that of all the lateral inter-radials amphiarthroses. All the fin rays are embedded in a continuous membrane that limits dorso-ventral displacement between neighboring rays. This structural arrangement increases the efficiency of the percussive action in the water column exerted by the dorsal and ventral muscles of the fins.

In *M. aquila*, the number of radials per ray increases progressively from the anterior margin towards the central axis of the fin—up to a maximum of 25 elements—and then decreases symmetrically towards the posterior margin (Figure 1(A1)). In *R. asterias*, the central rays are the longest and contain the highest number of radials, while there are fewer radials in the anterior region, which is consistent with the smaller surface area (Figure 1(B1)). In this species, the length of the rays decreases regularly from the base of the pterygium to the fin margin. In contrast, in *M. aquila* the length of the radials from the medial line to the lateral margin remains relatively constant (Figure 1(A1)). Although no morphometric analysis was performed, the comprehensive dorso-ventral radiograph suggests that these structural differences represent stable and distinguishing features between the two species.

Microscopic observations at higher magnification also confirmed clear differences in radial morphology between the species. In *R. asterias*, each radial is reinforced by two robust calcified columns embedded in a single non-calcified cartilaginous cylinder. These columns remain unconnected in the central portion, but branch at both ends to support a compact, unitary disk. This configuration is consistently observed in the medial sector of the fin. At approximately two-thirds of the length of the fin ray, the two calcified columns within the more compliant cartilaginous cylinder begin to rotate under torsional loading and shift in the horizontal plane. This displacement is initiated by the splitting of the lateral disk and further promoted by the separation of the medial disk. The process culminates in the longitudinal splitting of the non-calcified cartilage cylinder, effectively doubling the number of rays (each of which now contains a single calcified column in the lateral region of the fin). This structural change leads to increase the lateral fin flexibility (Figure 1(B1–B4)).

In *M. aquila*, the pectoral fin is also supported by radially oriented rays; however, their internal architectures differ from each other. The radials consist of a three-dimensional network of small, calcified tiles located between two compact end plates. These tiles are smaller than those of *R. asterias*, and the overall structure remains the same along the entire length of the ray, with no structural transitions (Figure 1(A2,A3)). This morphology indicates a lower flexibility of the fins compared to *R. asterias*, supporting their functional specialization in percussive locomotion.

In *S. aurata*, as previous literature has shown, the pectoral fins consist of long, parallel rays embedded in a flat connective tissue membrane that is covered with skin on the outside. Each fin ray branches and tapers from the base to the fin tip. The skeletal structure of each ray consists of regularly aligned and cylindrical units of calcified bone matrix tight through a thin layer of uncalcified fibrous-connective tissue between the latter. The comparison with the SEM external morphology of the Chondrichthyes fins suggests an almost stiff, fibrous link between the aligned units with a scarce flexibility of the teleost *S. aurata* fin ray.

### 3.2. Head, Oral Cavity and Dentition

**Figure 2:**
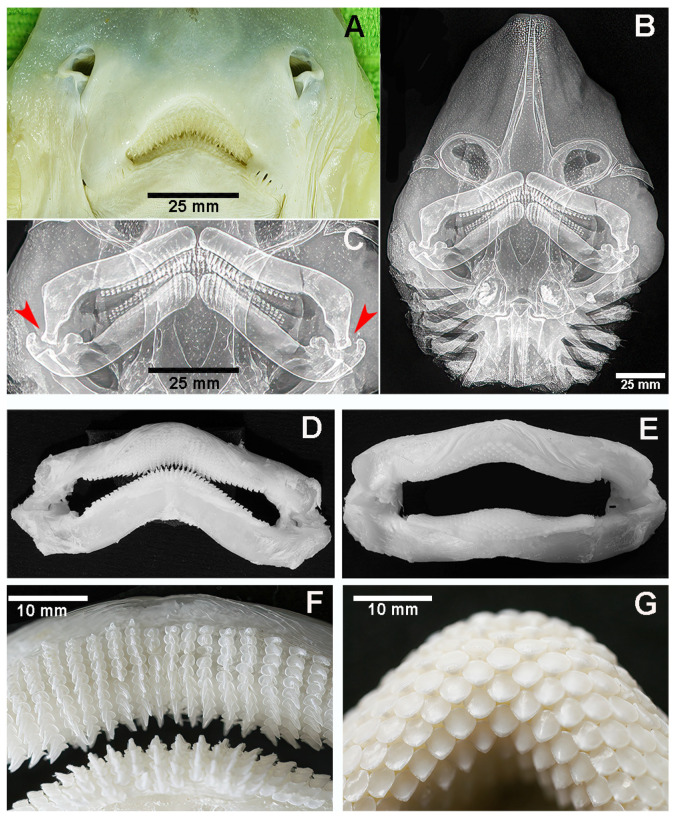
*Raja asterias*: external, ventral aspect of head and radiograph in dorso-ventral projection of mouth and teeth. (**A**) *R. asterias*: ventral view of nostrils and mouth of male specimen with pointed teeth. (**B**) Head dorso-ventral X-ray projection showing the pointed rostrum, the calcified elements of the head skeleton, with the thick upper and lower jaw and the gills. The diffuse, small white dots correspond to calcifications of the dorsal skin. (**C**) Dorso-ventral X-rays showing detail of the upper and lower jaw in a male specimen with pointed teeth. Both jaws are pulled together in the middle by a thick, fibrous ligament. The upper arch extremities bend symmetrically downwards, with the ball fitting into the socket (red arrows). (**D**,**E**) Maxillary and mandibular arches with pointed teeth in a male *R. asterias* specimen (**D**) and spherical teeth in a female (**E**). (**F**,**G**) Higher magnification of the teeth row pattern in a male (**F**) and female (**G**) *R. asterias*.

**Figure 3:**
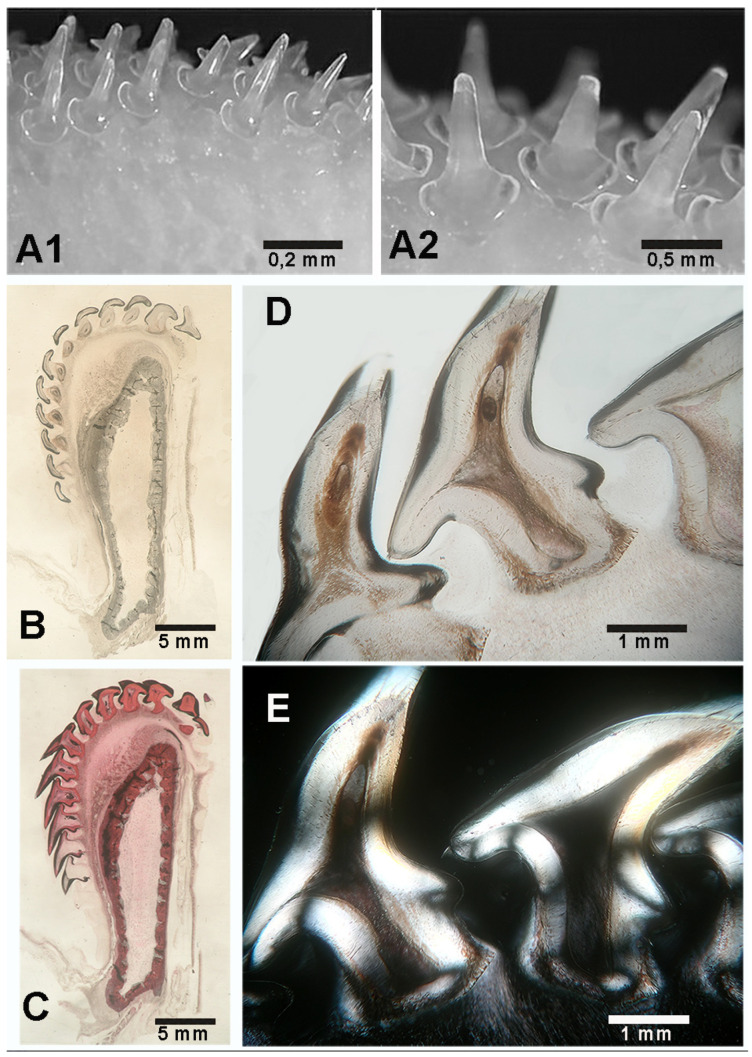
*Raja asterias*: details of male pointed teeth and their histology. (**A1**,**A2**) Enlarged base of pointed teeth leaning on the jaws surface. (**B**,**C**) Transverse, un-decalcified section of the lower jaw and teeth ((**B**) hematoxylin–eosin, (**C**) methylene blue–acid fucsine). The transversally sectioned teeth extend from the jaw upper edge to the anterior labial surface. The tessellated calcified cartilage pattern of the jaw wall surrounds the “core” of un-calcified cartilage. (**D**,**E**) Decalcified, teeth longitudinal sections at higher magnification (hematoxylin–eosin in bright field (**D**) and in polarized light (**E**), showing the pulp, the enlarged base leaning on the jaw surface and the tooth root deepening into the jaw calcified cartilage.

The head of *R. asterias* is flattened and triangular in shape. The calcified rostrum is covered by both the dorsal and ventral skin, which continues without interruption into the tegument of the left and right pectoral fins. Dorso-ventral X-ray projections show the characteristic inverted V-shape of the rostrum formed by two calcified elements that converge anteriorly into a single point. The mouth is located on the ventral surface (Figure 2A–C). Both the maxilla and mandible consist of two thick, calcified cartilaginous segments connected at midline by a robust fibrous band, forming the curved maxillary and mandibular arches. They are articulated at the lateral ends by ball-and-socket joints. The lateral ends of the upper jaw are bent downwards with an angle of approximately 70° so that the ball of the upper jaw fits securely into the corresponding socket of the lower jaw (Figure 2C). The teeth are distributed over most of the labial surface of both arches, with a lower density on the inner (lingual) surface (Figure 2D,F). The species exhibits sexual dimorphism in tooth morphology: Males have pointed teeth (Figure 2D,F), while females have spherical teeth (Figure 2E,G). Macroscopic details of the pointed teeth and their histological features in *R. asterias* are presented in Figure 3.

**Figure 4:**
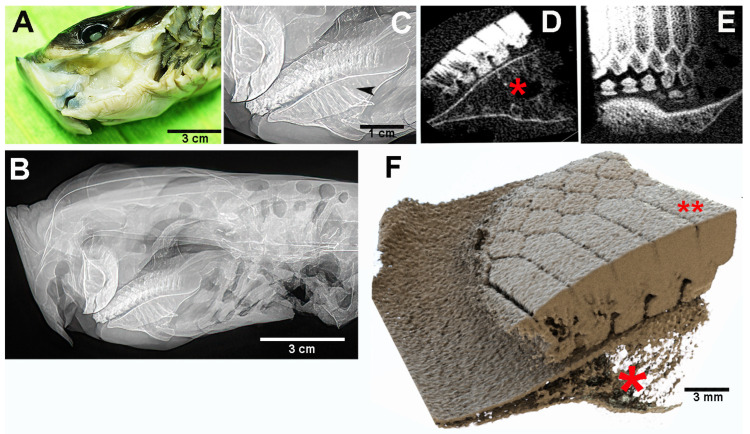
*Myliobatis aquila*: head and mouth external view, X-rays in lateral projection and micro-CT. (**A**) Oral cavity and gills exposed by dissection of the pectoral fin, the mouth opening at the anterior lower edge. (**B**,**C**) X-rays the lateral view of the head (**B**) and detail (**C**) of the symphyseal plates. The flat lower plate comes into contact with the curved upper plate in a narrow, anterior area. The latter are supported by vertical, calcified struts of Meckel’s cartilage segments and the palatal squares, without any contact with the mandibular arches. (**D**–**F**) Micro-CT shows the geometry and compactness of the symphyseal plates calcified cartilage. The latter lean on the cortex of the underlying skeletal elements (**D**–**F**), which are characterized by a poor calcification density (red asterisk). Load transmission is made on the cortex through distinct, cubic blocks (**F**). The higher compactness of the plates calcified cartilage is well documented ((**D**–**F**) red asterisk). A thin un-calcified cartilage layer (**D**–**F**) separates the symphyseal plate from the below skeletal elements (**D**–**F**).

**Figure 5:**
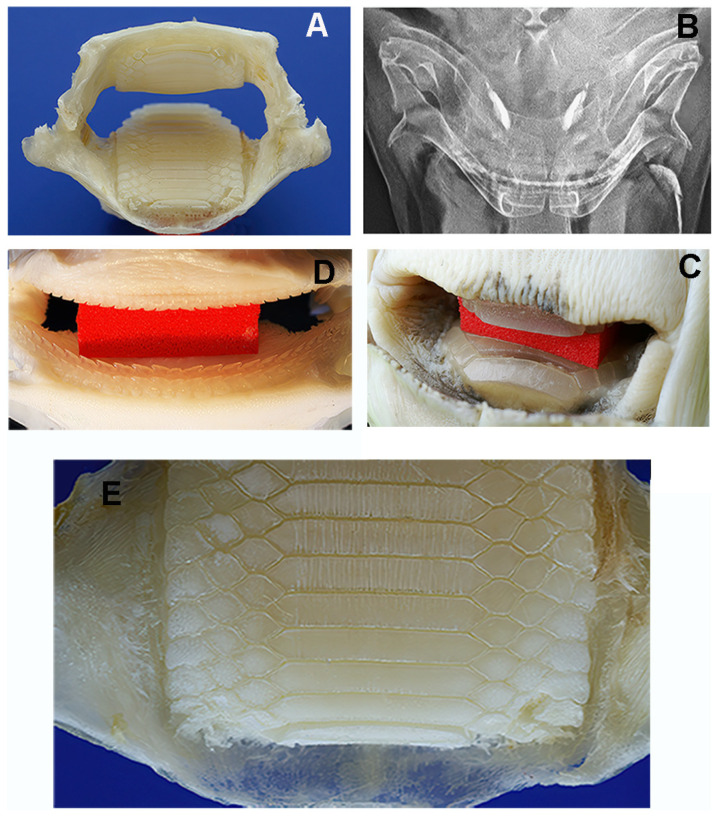
*Myliobatis aquila*: morphology of the double masticatory system. (**A**,**B**) Dissection of maxilla and mandible before removal of the symphyseal plates (**A**) and X-rays of the head in dorso-ventral projection (**B**). The upper and lower jaw (thinner than that of *R. asterias*) are external to the symphyseal plates. The movement of the system (adduction/abduction) is based on the ball-socket joints at the extremities. (**C**) Red spacer inserted between the upper and lower plates. (**D**) Full view of aligned teeth lines on the mandibular arches after red spacer removal. (**E**) Detail of the longitudinal scratches on the lower plate corresponding to the contact zone (anterior) with the curved upper plate.

**Figure 6:**
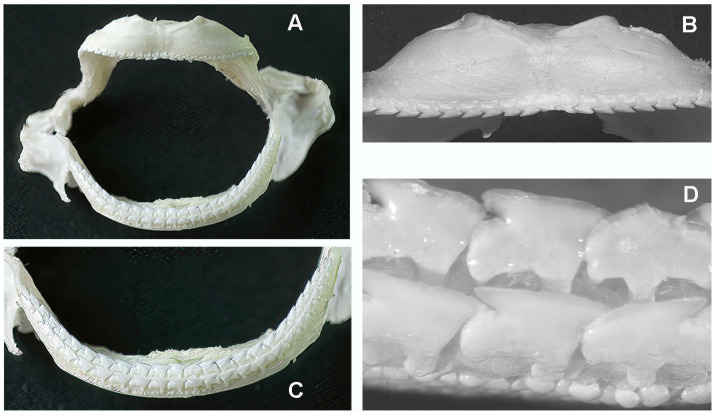
*Myliobatis aquila*: details of upper and lower jaws (with teeth) after removal of the internal symphyseal plates. (**A**) The thin maxillary and mandibular arches articulate at the right and left extremities with ball-socket joints. (**B**) Frontal view of the upper jaw with a single row of aligned, pointed teeth bending in opposite directions from the mid-central arch. (**C**) Lower jaw arch showing the two longitudinal, parallel teeth rows of aligned, pointed teeth. (**D**) Higher magnification of the teeth morphology shows the difference in shape and row orientation with the *R. asterias* pointed teeth. The line of small spherical bodies nearby the teeth corresponds to the skin surface.

In contrast, the head of *M. aquila* is squat and cuboidal, with a flat forehead and the mouth opening at the antero-ventral margin (Figure 4A). Two different dental systems are present inside the oral cavity: (a) an internal system consisting of upper and lower plates, referred to as “dental plates” [10,11] or “symphyseal p2lates” [8], which is a characteristic of durophagous Chondrichthyes (Figure 4B,C and Figure 5); and (b) an external system consisting of maxillary and mandibular arches supporting teeth, as in all Gnathostomata. Compared to the similarly heavy specimen of *R. asterias*, the jaws of *M. aquila* are longer, thinner and less robust (Figure 6A). A quantitative comparison of the jaw cross-sectional area and the outer surface confirms that *M. aquila* has significantly smaller dimensions, indicating a lower mechanical rigidity compared to *R. asterias*. However, the overall pattern of tooth distribution is similar in both species.

The symphyseal plates in *M. aquila* (Figure 4) are easily detached from the overlying calcified palatal cartilage and the underlying elements derived from Meckel’s cartilage. Dissection revealed that the thickness of the upper and lower plates (at full adduction) exceeds the height of the outermost rows of teeth bordering the jaw, suggesting that the plates are the primary contact surfaces during biting (Figure 5). The occlusal surface of the symphyseal plate system is structured with a dense central band of horizontal rectangular units bordered laterally by three rows of interlocking hexagonal units (Figure 4D–F). Lateral projections of the head show that the lower plate is almost flat, while the upper plate is dorsally concave, with a radius of curvature less than half the length of the lower plate (Figure 4B,C). This geometry indicates that the highest compressive forces occur within a narrow contact zone between the plates (Figure 5A–C). This statement is supported by the presence of regular, antero-posterior, parallel scratches on the occlusal surfaces—evidence of wear patterns typical of durophage feeding (Figure 5D). Micro-computed tomography (micro-CT) images of the symphyseal plates (Figure 4D–F) also show that mechanical forces are transmitted from the calcified cartilaginous skeleton to the occlusal surface via discrete, cube-shaped blocks (tesserae) of calcified cartilage (Figure 4D,F).

### 3.3. Morphology of the Non-Appendicular Skeleton in Chondrichthyes

The skeleton of Chondrichthyes is characterized by a peripheral, mosaic-like organization commonly referred to as “crustal pattern”. This term was introduced by Summers [11] and further elaborated by Schaefer & Summers [12]. Beneath the symphyseal plates, this structure is reinforced by internal calcified struts that support durophage loading (Figure 4B,C) and exhibit the same histomorphological features as the calcified columns observed in the radialis.

The maxillae and mandibles of *R. asterias* and *M. aquila* exhibit the typical skeletal architecture of chondrichthyans: a central core of non-calcified cartilage surrounded by a layer of calcified, polygonal cartilaginous cusps that form the crustal pattern. In *M. aquila*, however, the entire jaw structure is significantly thinner and less rigid than in *R. asterias*. This is a morphological impression suggesting lower rigidity, rather than a quantified result.

Although only a limited number of *R. asterias* specimens were examined, a clear sexual dimorphism was observed: males had pointed teeth, whereas females had spherical teeth (Figure 2C–E). In contrast, all three examined specimens of *M. aquila* had pointed teeth, so that no conclusion can yet be drawn regarding a possible sexual dimorphism in this species. In both species, the teeth—whether pointed, spherical or of another shape—are arranged in regular, parallel rows: perpendicular to the jaw axis in *R. asterias* and longitudinal in *M. aquila* (Figure 6B,C).

**Figure 7:**
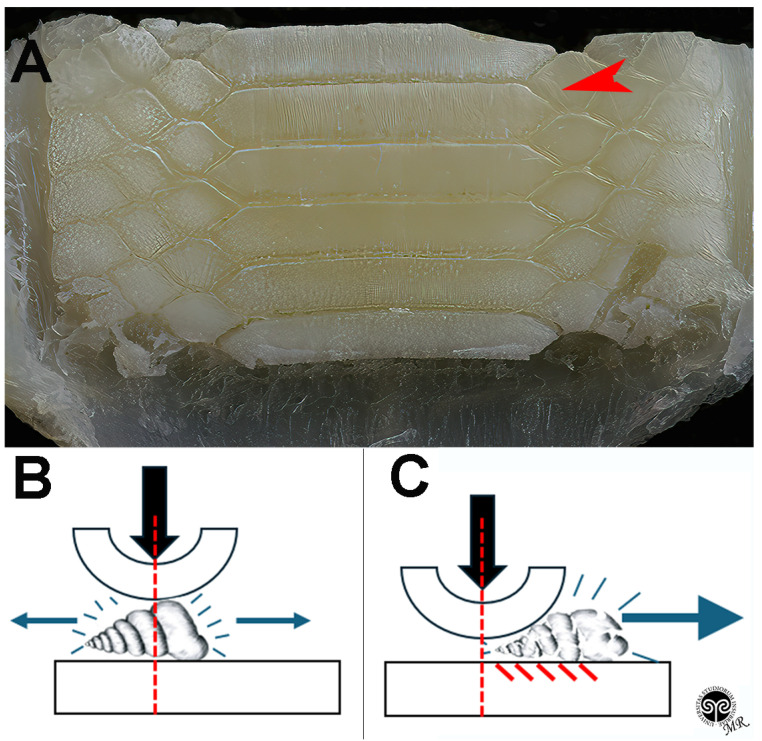
*Myliobatis aquila*: crushing mechanism of the symphyseal plates. (**A**) Elongated, parallel scratches are evident on the lower plate dorsal surface, where jaws adduction develops the highest compressive force with the curved, upper plate. (**B**,**C**) Shell fragments can be pushed forward or backward to the digestive tract by the same compressive force or by the hydrodynamic water flow in the mouth.

In *R. asterias*, teeth are present on both the apical and frontal surfaces (Figure 3C). Each *R. asterias* tooth (male and female) has a broad, rounded base resting on the maxillary surface (Figure 2F,G), with a short cylindrical root embedded deeper in the underlying cartilage (Figure 3D,E). The spherical teeth are shorter and have a different distribution pattern from the pointed teeth, whose view from above suggests an occlusal-like surface, though not as compact as the symphyseal plates (Figure 4B,C). The crushing mechanism of the symphyseal plates in *M. aquila* is shown in Figure 7.

### 3.4. Scanning Electron Microscopy and Mineral Analysis of the Pectoral Fins in Chondrichthyes and Teleosts

A comparative SEM analysis of the pectoral fins of *M. aquila*, *R. asterias* and the teleost species *S. aurata* was performed. According to our observations, the pectoral fin tiles of *M. aquila* and *R. asterias* showed no substantial differences in matrix composition or ultrastructure, apart from variations in tile size and column morphology, which were already evident under light microscopy. In contrast, the cylindrical elements of the fin radials in the teleost *S. aurata* served as a useful model for comparing the morphology and mineral composition of the calcified matrix between Chondrichthyes and Actinopterygii.

**Figure 8:**
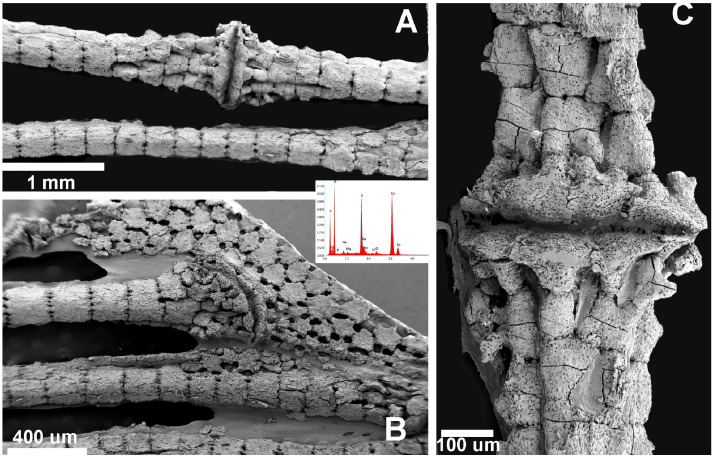
*Raja asterias*: SEM-morphology of fins and joints and EDAX analysis of the mineralized phase. (**A**) Radials’ columnar pattern and inter-radials joint: EDAX analysis documenting Ca_3_(PO_4_)_2_ spectrum. (**B**) Tesseral “mosaic” pattern of pterygium and pterygial-first radial joint. (**C**) Detail of inter-radial joint showing the joint disk plate and the chondrocyte lacunae opening on the external surface of the calcified cartilage elements.

In *R. asterias*, SEM analysis of the pectoral fin radials revealed the characteristic columnar arrangement of calcified cartilage, in which elongated cylindrical tiles are aligned along the longitudinal axis of the ray (Figure 8A). These columns are interconnected at their extremities by thin joint disks that allow limited flexibility while maintaining overall structural stiffness. In contrast, the pterygial region and the proximal joints displayed the typical tessellated “mosaic” pattern of flat, polygonal tiles (Figure 8B), consistent with the crustal architecture that supports load distribution across the basal fin skeleton. Together, these images document the coexistence of two distinct skeletal layouts within the same fin: columnar organization in the distal radials, promoting directional strength, and tesseral organization in the basal elements, enhancing resistance to multidirectional stresses.

**Figure 9:**
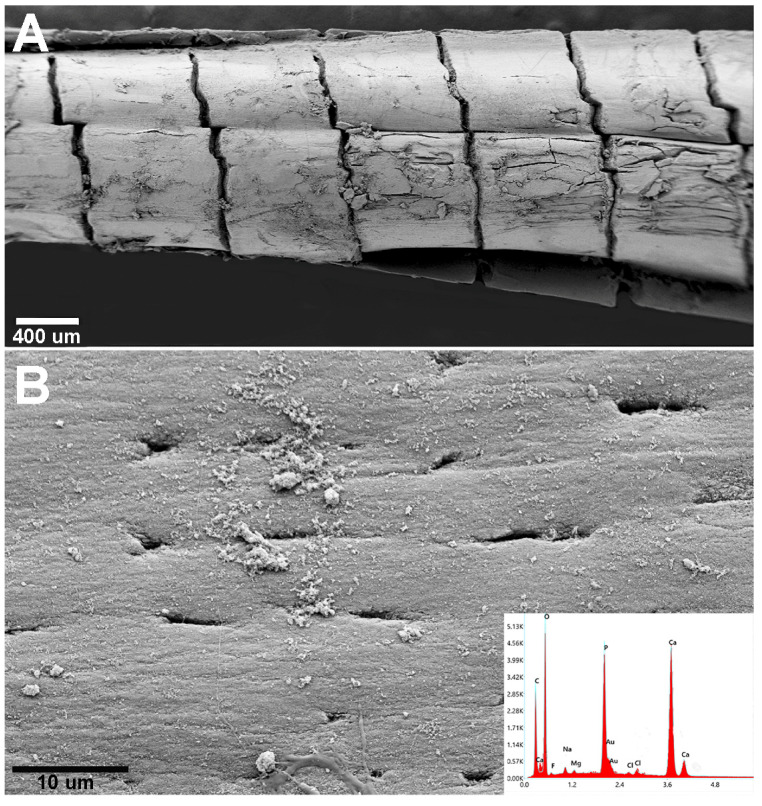
*Sparus aurata* (teleost) pectoral fin. (**A**) SEM morphology with aligned cylindrical units with a compact, smooth surface. (**B**) Few, flat osteocyte lacunae open on the external surface and EDAX confirming Ca_3_(PO_4_)_2_ spectrum of the calcified bone matrix.

In the chondrichthyans, the radial columns consist of aligned, cylindrical calcified tiles that branch at the ends to support the inter-radial articular disks (Figure 1(B2,B4)). Other skeletal segments—such as the pterygia and girdle—are composed of flat, polygonal plates characteristic of the crustal pattern (Figure 8B). At higher magnification, the calcified matrix of Chondrichthyes reveals the structure of the inter-radial joint, with the joint disk plate clearly visible and chondrocyte lacunae opening onto the external surface of the calcified cartilage elements (Figure 8C). The pectoral fin rays of the teleost *S. aurata* are also composed of aligned cylindrical units (Figure 9A); however, the calcified intercellular matrix is more compact and presents a lower density of flattened osteocyte lacunae, as shown by SEM (Figure 9B).

X-ray energy-dispersive spectroscopy (EDAX) elemental analysis identified the mineral phase in both the Chondrichthyes and Teleost specimens as calcium phosphate [Ca_3_(PO_4_)_2_] (Figure 8 and Figure 9). These results provide evidence that the mineral phase deposited in the extracellular matrix is consistently Ca_3_(PO_4_)_2_, regardless of whether the matrix is synthesized by chondrocytes (in Chondrichthyes) or osteoblasts (in Teleosts).

**Figure 10:**
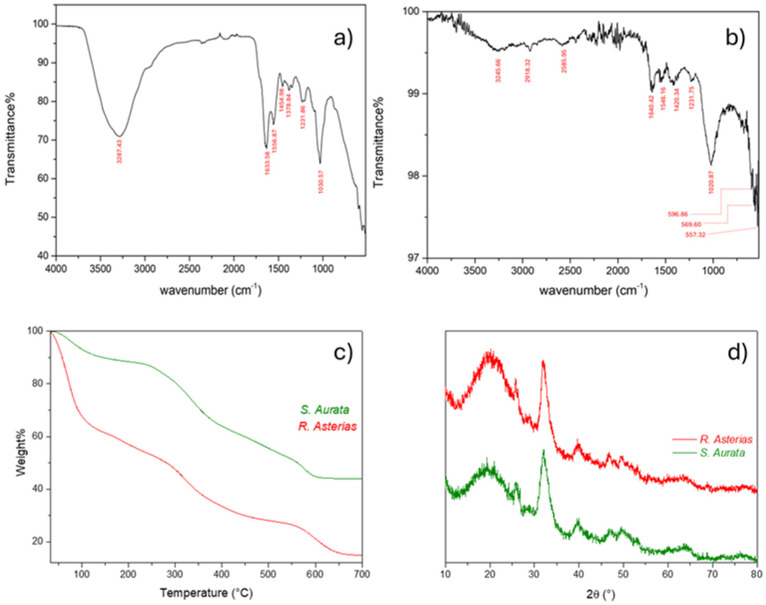
Physicochemical comparative analyses of fins from *S. aurata* (Teleost) and *R. asterias* (Chondrichthyes): (**a**) FT-IR spectrum of *S. aurata*; (**b**) FT-IR spectrum of *R. asterias*; (**c**) thermogravimetric curves related to *S. aurata* (green) and *R. asterias* (red); (**d**) XRD pattern comparison between *R. asterias* (red) and *S. aurata* (green). No significant (or minimal) differences were observed between the Ca_3_(PO_4_)_2_ mineral phase in the Teleost bone matrix and the calcified cartilage matrix of the Chondrichthyes, indicating a comparable mineral deposition.

Physicochemical analyses were conducted to compare the fins of the teleost *S. aurata* and the batoid *R. asterias*. The thermogravimetric analysis (TGA) profiles (Figure 10c) showed similar thermal degradation patterns in both species, with overlapping temperature-dependent transitions. Fourier-transform infrared spectroscopy (FTIR) (Figure 10a,b) confirmed the presence of phosphate vibrational bands characteristic of calcium phosphate mineralization in both matrices.

The temperatures, percentages of mass loss, and corresponding phenomena from the TGA analyses are shown in Table 2. Overall, these results indicate that no significant differences exist between the mineral phase of the teleost bone matrix and that of the calcified cartilage in the batoid, suggesting a comparable mineral deposition process.

From the physicochemical characterization, several assumptions can be made regarding the differences and similarities between the two batoid species, *R. asterias* and *S. aurata* (the characterization of *M. aquila* is reported in the Supplementary Materials, Figures S3–S5, and Table S1).

XRD analyses revealed no significant or only minimal differences between the Ca_3_(PO_4_)_2_ mineral phase present in the Teleost bone matrix and that of the calcified cartilage of the Chondrichthyes, indicating a comparable degree of mineral deposition. A similar pattern was observed in the TGA analyses, where both species displayed three main weight-loss steps associated with the release of water molecules, the thermal degradation of proteoglycans, and the initial partial decomposition of the collagen matrix. *R. asterias*, after treatment, had 15.05% residual material, attributed to remaining matrix due to residual collagen and inorganic salts, while *S. aurata* retained 44.03% residual matrix due to undegraded collagen and hydroxyapatite.

Despite these similarities in structural and thermal behavior, FT-IR fingerprints highlighted a marked difference between the two species (Figure 10a,b). The spectrum of *R. asterias* appeared smoother, with distinct and well-resolved bands, whereas that of *S. aurata* exhibited a noisier and more irregular profile. This behavior indicates a higher content of apatite and hydroxyapatite minerals in the Teleost sample. The crystallization of these mineral phases into rigid, anisotropic grains leads to light scattering effects, resulting in a non-uniform and fluctuating baseline in the ATR spectrum—an effect not observed for *R. asterias* (Figure 10a).

Additionally, in *R. asterias*, characteristic bands associated with cartilaginous matrices were clearly detected, such as the strong –OH stretching peak at 3287.43 cm^−1^, which appeared only weakly in *S. aurata* (Figure 10b), and the –PO_4_^3−^ vibrations in the 500–600 cm^−1^ region, evident in the Teleost but not appreciable in the Chondrichthyan spectrum.

## 4. Discussion

This study compared the morpho-anatomy of two batoid species (*R. asterias* and *M. aquila*) to document how ontogeny and cartilage mineralization have evolved to adapt the pectoral fins and dentition to their respective locomotion mechanics and feeding strategies. The evolution of pectoral fins morphology and function has been extensively studied by different research groups [12,13,14,15,16], while dentition and durophagy have been studied by Summers [11] and a wide literature can be found on sexually dimorphic dentition and feeding adaptations in Chondrichthyans [8,17,18,19,20,21,22,23,24,25].

(A) Pectoral fins: Development, structure and functional adaptation.

During the development of the vertebrate skeleton, the cartilage differentiates early from the mesenchyme and is subsequently stiffened by the deposition of minerals. In most vertebrates, this mineralized cartilage is later replaced by bone through endochondral ossification, which involves the following: Chondroclast-mediated resorption, i.e., “modeling” of developing bone, and osteoblast-mediated deposition, i.e., “remodeling” of the adult skeleton—two processes that are well documented in mammals and *Homo sapiens* [3,26]. Remodeling also allows fractured bones to heal with the unique ability to restore the original trabecular and osteonal architecture while maintaining the mechanical performance of the skeletal segment.

Unlike endochondral bone, the calcified cartilage of Chondrichthyes (subdivision Batoidea) stiffens primarily through the appositional mineralization of tesserae and does not undergo secondary, Haversian-type osteonal remodeling [27]. Teleost fishes, by contrast, display both cellular and acellular bone, depending on the taxon and the specific skeletal element [28]. In our *S. aurata* specimens, SEM analysis revealed flattened osteocyte lacunae within the fin rays, which is consistent with the presence of cellular bone. It should be emphasized, however, that teleost skeletal tissues are heterogeneous, and acellular bone is well documented in other species and anatomical structures [28].

With respect to Chondrichthyes (subdivision Batoidea), the ontogeny of the appendicular skeleton and the morpho-anatomy of *R. asterias* and *M. aquila* reveal two different strategies to modulate fin flexibility:

1. Segmentation through the development of cartilage radii “anlagen” along the fin rays, which are controlled by key developmental gene families involved in limb and fin formation [29].
2. Anatomical distribution of mineral deposits that influences radial stiffness both between species and between fins within the same species [30].

In *M. aquila*, the pectoral fin has a uniformly rigid structure along all radials, characterized by a uniform 3D mineral network extending from the medial pterygial base to the lateral fin margin. Restricted inter-radial mobility due to the narrow amphiarthroses results in limited flexibility of the fin—an arrangement suitable for flapping locomotion in the water column.

In contrast, *R. asterias* has a more complex pectoral fin architecture. The medial radial fins consist of two firmly connected, calcified columns, while the lateral radial fins become increasingly flexible due to the splitting of the terminal disks and the longitudinal splitting of the central cartilaginous cylinder. This structural transformation allows the columns to rotate horizontally and creates two independent, single-column rays, effectively doubling the number of rays and increasing the flexibility of the fins. This morphology supports the fluttering, undulating locomotion of the species [27].

Ecological data confirm the observed morphological and functional differences between the two species. *M. aquila* is a benthic-pelagic species that typically lives in deeper waters than *R. asterias* [25,31,32,33,34]. Its flapping locomotion, supported by uniformly stiff pectoral fins, is well adapted to sustained swimming in the water column and allows the species to cover long distances and maintain buoyancy in less structured, open environments.

In contrast, *R. asterias* inhabits shallow, sandy seabeds, where it feeds on softer benthic organisms. Its fluttering, undulating swimming mode, enabled by a pectoral fin structure that is rigid in the middle and increasingly flexible laterally, allows for precise maneuverability near the substrate. This swimming strategy provides a functional advantage in benthic habitats, where subtle, localized movements facilitate efficient foraging and navigation through complex or unstable sedimentary environments.

(B) Head/mouth and teeth ontogeny. The head morphology and the morphoanatomy of the oral and dental structures showed significant differences between *M. aquila* and *R. asterias*. These differences provide valuable insights into the evolutionary adaptation of the cranial skeleton and dentition to the feeding strategies and ecological niches occupied by the two species. The present study focused on two main aspects:

1. Durophagy—comparison of the mouth and dentition of *M. aquila* and *R. asterias*;
2. Sexual dimorphism—analyzing the differences in dental morphology between male and female *R. asterias*.

### 4.1. Durophagy and Jaw Mechanics

Despite differences in head shape—broad, flat, and rounded with a straight forehead in *M. aquila*, and flat and triangular with a pronounced frontal rostrum in *R. asterias*—the upper and lower jaws of both species are similar in structure. However, *R. asterias* exhibits a mandibular structure that appears more robust and rigid, consistent with its substrate-oriented feeding habits. We emphasize that this is a morphological observation indicating greater rigidity, rather than a result derived from our quantitative measurements.

In *M. aquila*, the inner mass of the symphyseal plates occupies most of the oral cavity, while the slender maxillae and mandibles—bearing teeth of a different shape—are positioned outside the plates. It is noteworthy that the thickness of the symphysis plates exceeds the height of the maxillary and mandibular teeth at full adduction, which prevents occlusion between the opposing rows of teeth. As far as can be seen from the current literature, this anatomical configuration is unique and may indicate that *M. aquila* has evolved two distinct masticatory systems:

- An external system consisting of the maxillary and mandibular arches and their associated teeth.
- An internal system consisting of the thick, calcified symphyseal plates specialized for crushing hard-shelled or exoskeleton-bearing organisms, such as corals, shelled mollusks, or crabs.

Alternatively, if we reject the concept of dual masticatory systems, we could hypothesize that the ancestral teeth have been preserved but no longer function due to interference with the symphyseal plates, possibly performing residual or other structural functions.

The mechanisms of durophagy in the Myliobatoidea were studied by Summers [11] in *Rhinoptera bonasus*, whose symphyseal plates are similar to those of *M. aquila*. According to our findings, the plates function by pressure and shearing movements generated by the mouth muscles. The lower plate is almost flat, while the upper plate is remarkably curved and opens dorsally. Contact between the plates occurs in a narrow, transverse area located anteriorly on the lower plate (visible in the lateral radiographs) and serves as a focal point for the maximum compressive force.

The documented wear patterns—especially the longitudinal scratches, which are limited to the high-pressure area—indicate that the shell fragments of the mollusk prey are pushed either forwards or backwards after fragmentation. These fragments are probably transported into the digestive tract either by the pressure force itself or by the hydrodynamic flow during suction feeding.

A central question is whether the movement of the jaws is mechanically linked to the movement of the symphyseal plates [8,35,36]. The euhyostylic jaw suspension typical of batoids in combination with highly complex cranial musculature [37] makes it difficult to identify independent muscular control over the two systems. However, dissection of our specimens revealed that the symphyseal plates are not structurally supported by the maxillary or mandibular arches, but are instead anchored to the calcified cartilage of the palatine bones and Meckel’s cartilage. These findings support the hypothesis that separate muscle systems may control the internal (durophage) and external (biting or grasping) masticatory apparatus.

### 4.2. Sexual Dimorphism and Dental Variability

Tooth shape, arrangement and function are not fixed features in batoids. Differences are observed not only between species but also within species depending on sex, age and seasonal dynamics. In *R. asterias*, sexual dimorphism is evident in the dentition: males have pointed teeth adapted to feeding and grasping, while females have shorter, spherical teeth that are more densely arranged, resulting in a more uniform chewing surface.

Similar patterns have also been observed in other chondrichthyans. In *Hypanus sabinus* (Lesueur 1824), for example, Kajiura & Tricas [19] found that the teeth of females have a stable, molariform shape, except during the breeding season when they adopt a more pointed morphology—presumably for mating purposes. In males, the pointed teeth are used to grip females during copulation, an adaptation that is also well documented in Rajidae [17,18,38,39].

This dental plasticity illustrates the evolutionary adaptability of the feeding apparatus to the demands of the environment and reproduction. The existence of two functionally distinct masticatory systems in *M. aquila*, as proposed in this study, may also be supported by the complex cranial musculature and angular cartilage structures [40] observed in this group. Further evidence comes from an analysis of the feeding habits of 273 specimens of the skate (*Dipturus laevis*), which found significant differences in prey size, shell hardness and crustacean type that correlated with tooth morphology and sexual dimorphism [41].

### 4.3. Mineralization Processes in Skeletal Tissues

The physicochemical analyses conducted in this study (TGA, DSC, FTIR, and XRD; Figure 10 and Table 2) showed that both the calcified cartilage of R. asterias and the bony fin rays of *S. aurata* share the same mineral phase, consisting of calcium phosphate (Ca_3_(PO_4_)_2_). The thermogravimetric curves indicated similar temperature-dependent mass loss transitions, while FTIR spectra revealed identical phosphate vibrational bands. XRD also confirmed the crystalline phase as Ca_3_(PO_4_)_2_ in both taxa. These findings provide the experimental basis for interpreting mineralization strategies in chondrichthyan and teleost skeletal tissues.

It is important to note that the mechanisms underlying skeletal mineralization are well established in the literature and are not presented here as new discoveries. Specifically, spontaneous precipitation of calcium salts alone does not account for the organized mineral deposition observed in vertebrate skeletal tissues. Enzymatic activity, such as that of alkaline phosphatase, and the involvement of extracellular matrix vesicles have been shown to play essential roles in initiating and controlling crystal nucleation and growth [42,43].

Within this framework, our findings indicate that, although batoids and teleosts differ in skeletal tissue type—calcified cartilage in R. asterias versus cellular bone in *S. aurata*—the mineralization processes converge on the deposition of the same calcium phosphate phase. This suggests that despite evolutionary divergence in skeletal architecture, the biochemical pathways leading to mineral phase formation are conserved. Our results therefore help bridge the gap between structural diversity and shared mineralization strategies across vertebrate lineages.

## 5. Conclusions

In summary, the hitherto unknown presence of two different chewing systems in *M. aquila* documents an evolutionary adaptation to nutritional requirements that can be explained by two hypotheses: the coexistence of two functioning systems in current specimens, allowing for the ingestion of harder and softer prey (or plant food), or the persistence of a rudimentary dentition that is no longer used (vestigial dentition). Furthermore, the texture of the calcified matrix in teleost fishes, as observed by SEM, may indicate a bone-like organic matrix substrate, similar to that found in endochondral ossification.

## Figures and Tables

**Table 1:** Data of the examined specimens (Chondrichthyes *R. asterias*, *M. aquila* and Teleost *S. auratus*.

nr.	Species	Sex	Weight (g)	Length (cm)
1	*R. asterias*	M	740	45
2	*R. asterias*	F	450	35
3	*R. asterias*	F	330	30
4	*M. aquila*	M	710	35
5	*M. aquila*	F	370	29
6	*S. aurata*	M	390	32
7	*S. aurata*	M	420	43

**Table 2:** Attributed transitions to the mass losses observed in the TGAs of Figure 10 for both *R. asterias* and *S. aurata* (DTG curves are reported in Figures S1 and S2 for clarification).

***S. aurata***		
Temperature (°C)	Mass loss	Attributed transition
RT-150	38.8	Adsorbed H_2_O molecules, loosely bounded H_2_O (superficial and interstitial)
150–530	32.87	Decomposition of the organic matrix (proteoglycans)
530–700	13.28	Decomposition of collagen matrix
***R. asterias***		
Temperature (°C)	Mass loss	Attributed transition
RT-200	11.65	Adsorbed H_2_O molecules, loosely bounded H_2_O (superficial and interstitial)
200–540	36.86	Decomposition of the organic matrix (proteoglycans)
540–700	8.257	Decomposition of collagen matrix

## Data Availability

All the data are presented in the article.

## Supplementary Materials

The following supporting information can be downloaded at https://www.mdpi.com/article/10.3390/ani15203034/s1, Table S1. List of temperatures, mass losses and attributed transitions observed in Figure S3. Figure S1. Complete TGA analysis of *R. asterias*. The black curve represents the mass loss in time, while the blue curve represents the DTG curve. Figure S2. Complete TGA analysis of *S. aurata*. The black curve represents the mass loss in time, while the blue curve represents the DTG curve. Figure S3. Complete TGA analysis of *M. aquila*. The black curve represents the mass loss in time, while the blue curve represents the DTG curve. This specimen shown a 59.59% residual matrix due to residual collagen and mineralised matrix (apatites, carbonates). Figure S4. IR spectrum of *Myliobatis aquila*. Figure S5. XRD pattern of *M. aquila*. The material is completely amorphous.
