# Supplementary material for: Skeletal Adaptations to Locomotion and Feeding in Mediterranean Batoids (Raja asterias, Myliobatis aquila) and the Teleost Sparus aurata: A Comparative Study
Source: Animals (Basel). 2025 Oct 19;15(20):3034. doi: 10.3390/ani15203034 (PMC12561756; doi:10.3390/ani15203034)
Supplement: Supplementary file 1 [file animals-15-03034-s001.zip › animals-3889951-supplementary.pdf]

Skeletal Adaptations to Locomotion and Feeding in Mediterranean Batoids (*Raja asterias*, *Myliobatis aquila*) and the Teleost *Sparus aurata*: A Comparative Study

Ugo E. Pazzaglia [1,2,*], Genciana Terova [3,*], Marzia Guerrini [4], Piero A. Zecca [2], Guido Zarattini [1], Fabrizio Serena [5], Cecilia Mancusi [6] and Marcella Reguzzoni [2]

1 Department of Medical and Surgical Specialties, Radiological Sciences and Public Health, University of Brescia, 25121 Brescia, Italy; guido.zarattini@unibs.it
2 Department of Medicine and Technological Innovation, University of Insubria, 21100 Varese, Italy; pieroantonio.zecca@uninsubria.it (P.A.Z.); marcella.reguzzoni@uninsubria.it (M.R.)
3 Department of Biotechnology and Life Sciences, University of Insubria, 21100 Varese, Italy
4 Department of Chemistry, Physical Chemistry Section, C.S.G.I. (Consorzio Inter Universitario per lo Sviluppo dei Sistemi a Grande Interfase), University of Pavia, 27100 Pavia, Italy; marzia.guerrini01@universitadipavia.it
5 Institute of Marine Biological Resources and Biotechnology, National Research Council (CNR-IRBIN), 91026 Mazara del Vallo, Italy; fabrizio50serena@gmail.com
6 Environmental Protection Agency of Tuscany Region (ARPAT), 57126 Livorno, Italy; c.mancusi@arpat.toscana.it
* Correspondence: u.e.pazzaglia@gmail.com (U.E.P.); genciana.terova@uninsubria.it (G.T.); Tel.: +39-0332-421-428 (G.T.)

Supplementary Information

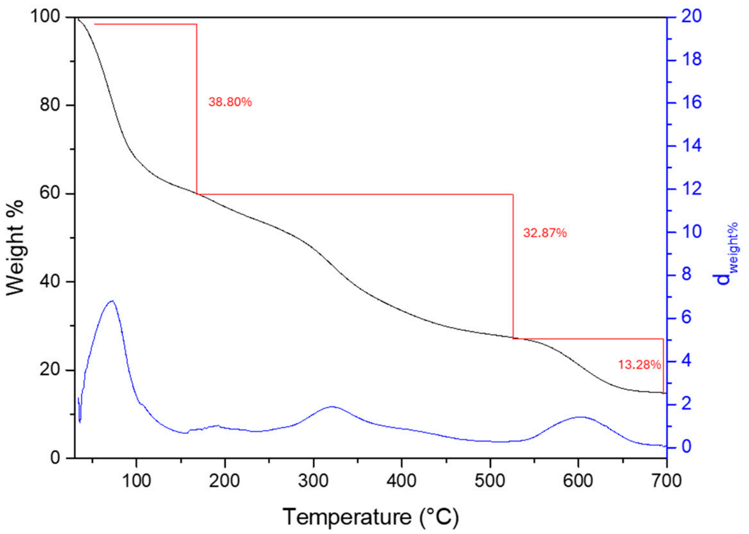

Figure S1. Complete TGA analysis of *R. Asterias*. The black curve represents the mass loss in time, while the blue curve represents the DTG curve.

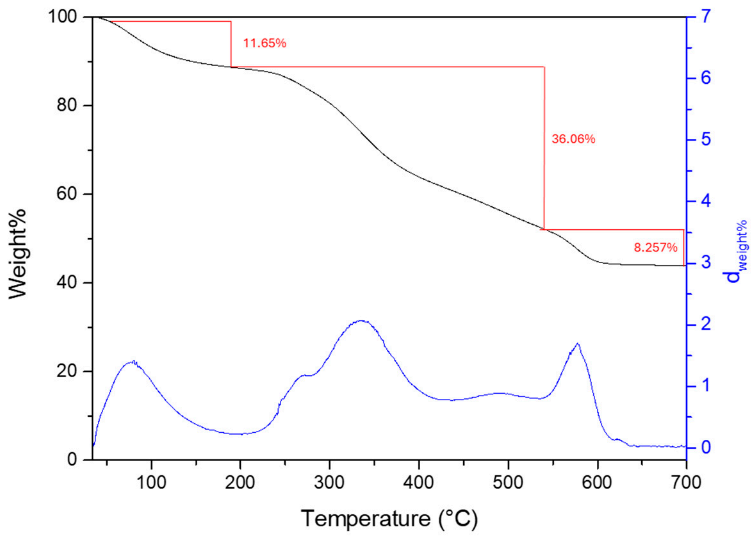

Figure S2. Complete TGA analysis of *S. Aurata*. The black curve represents the mass loss in time, while the blue curve represents the DTG curve.

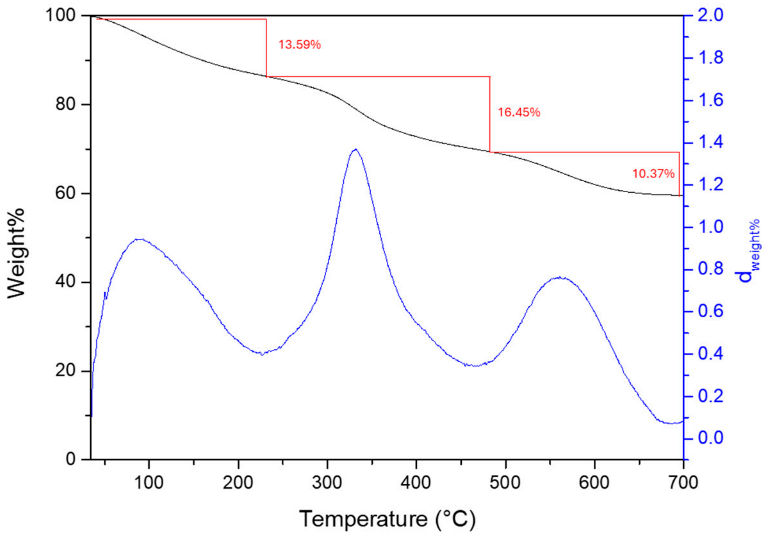

Figure S3. Complete TGA analysis of *Myliobatis aquila*. The black curve represents the mass loss in time, while the blue curve represents the DTG curve. This specimen shown a 59,59% residual matrix due to residual collagen and mineralised matrix (apatites, carbonates).

Table S1. List of temperatures, mass losses and attributed transitions observed in Figure S3.

Temperature (°C)	Mass loss	Attributed transition
RT-240	38.8	Adsorbed H_2O molecules, loosely bounded H_2O (superficial and interstitial)
240-460	32.87	Decomposition of the organic matrix (proteoglycans)
460-700	13.28	Decomposition of collagen matrix

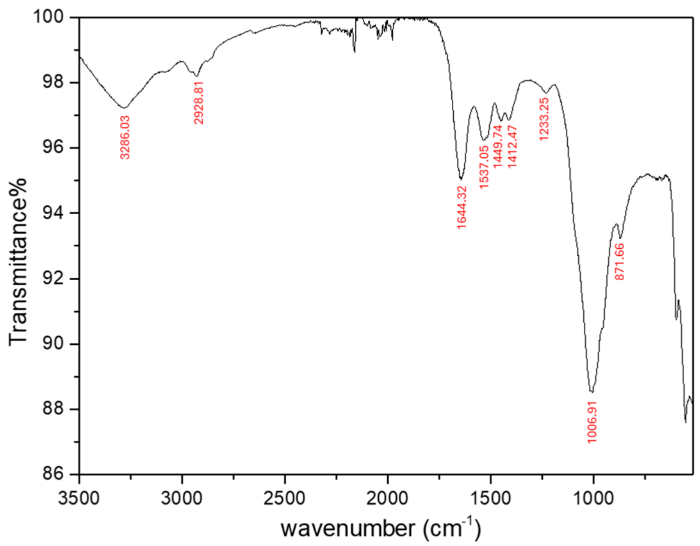

.

Figure S4. IR spectrum of *Myliobatis aquila*.

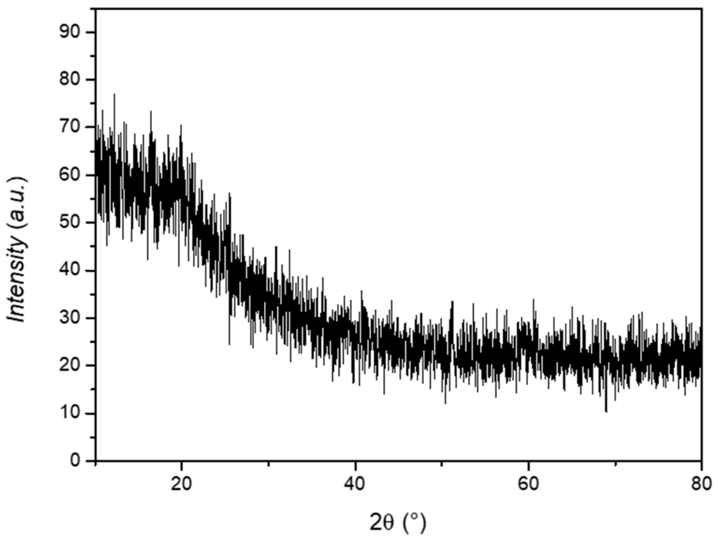

Figure S5. XRD pattern of *Myliobatis aquila*. The material is completely amorphous.
